# Increased MiR-221 expression in hepatocellular carcinoma tissues and its role in enhancing cell growth and inhibiting apoptosis *in vitro*

**DOI:** 10.1186/1471-2407-13-21

**Published:** 2013-01-16

**Authors:** Minhua Rong, Gang Chen, Yiwu Dang

**Affiliations:** 1 Research Department, Affiliated Cancer Hospital, Guangxi Medical University, 71 Hedi Road, Nanning, Guangxi Zhuang Autonomous Region, 530021, P.R. China; 2 Department of Pathology, First Affiliated Hospital, Guangxi Medical University, 6 Shuangyong Road, Nanning, Guangxi Zhuang Autonomous Region, 530021, P.R. China

**Keywords:** MiR-221, Hepatocellular carcinoma (HCC), Cell growth, Cell cycle, Apoptosis, Caspase

## Abstract

**Background:**

MiR-221 is over-expressed in human hepatocellular carcinoma (HCC), but its clinical significance and function in HCC remains uncertain. The aim of the study was to investigate the relationship between miR-221 overexpression and clinicopathological parameters in HCC formalin-fixed paraffin-embedded (FFPE) tissues, and the effect of miR-221 inhibitor and mimic on different HCC cell lines *in vitro*.

**Methods:**

MiR-221 expression was detected using real time RT-qPCR in FFPE HCC and the adjacent noncancerous liver tissues. The relationship between miR-221 level and clinicopathological features was also analyzed. Furthermore, miR-221 inhibitor and mimic were transfected into HCC cell lines HepB3, HepG2 and SNU449. The effects of miR-221 on cell growth, cell cycle, caspase activity and apoptosis were also investigated by spectrophotometry, fluorimetry, fluorescence microscopy and flow cytometry, respectively.

****Results**:**

The relative expression of miR-221 in clinical TNM stages III and IV was significantly higher than that in the stages I and II. The miR-221 level was also upregulated in the metastatic group compared to the nonmetastatic group. Furthermore, miR-221 over-expression was related to the status of tumor capsular infiltration in HCC clinical samples. Functionally, cell growth was inhibited, cell cycle was arrested in G1/S-phase and apoptosis was increased by miR-221 inhibitor *in vitro*. Likewise, miR-221 mimic accelerated the cell growth.

**Conclusions:**

Expression of miR-221 in FFPE tissues could provide predictive significance for prognosis of HCC patients. Moreover, miR-221 inhibitor could be useful to suppress proliferation and induce apoptosis in HCC cells. Thus miR-221 might be a critical targeted therapy strategy for HCC.

## Background

Primary liver cancer includes hepatocellular carcinoma (HCC), intrahepatic cholangiocarcinoma (ICC), and hepatic angiosarcoma. As the third leading cause of death from cancer (an estimated 549,000 deaths per year), HCC accounts for 85–90% of all primary liver cancers and ranks as the fifth most prevalent malignancy all over the world [1-4]. The development and progression of HCC is typical of a multistage process. It has been well documented that infection with hepatitis B and C virus (HBV and HCV) is the major etiological factor for the development of HCC [1-3,5,6]. The progression is considered to involve the deregulation of genes that are critical to cellular processes such as cell cycle control, apoptosis, cell migration and invasion. Many reports have highlighted on investigating genes and proteins underlying the development and progression of HCC, however, their sensitivity and specificity are limited [7-14]. Therefore, the identification of new biomarkers is urgently needed in order to understand the events causing hepatocarcinogenesis, also to relate various phenotypes in clinical features and prognosis and, more importantly, to predict response possibilities to therapeutic approaches.

The current therapies for HCC remain also challenging. At the earliest stages, HCC is treatable by resection or transplantation. Percutaneous ablation is an option in patients who are afflicted with early HCC and not suitable for resection or transplantation [15,16]. Transarterial chemoembolization has been effectively utilized in patients with HCC of intermediate stage [17]. Patients with advanced disease or whose cancer recurs following regional therapy have a dismal prognosis. New molecular therapies for HCC include epidermal growth factor receptor (EGFR) inhibitors, for instance, erlotinib [18] and antiangiogenic compounds, such as bevacizumab [19,20] and sunitinib [21]. In a phase III trial, patients with advanced HCC treated with the molecular targeted agent sorafenib, reported an increase in survival of approximately 3 months [22-25]. Nevertheless, new agents must be developed to treat advanced HCC.

In recent years, microRNAs (miRNAs) have received great attention in cancer research. These small, non-coding RNAs can inhibit target gene expression by binding to the 3’-untranslated region (3’-UTR) of target mRNA, resulting in either mRNA degradation or inhibition of translation to protein. MiRNAs play essential roles in many normal biological processes involving cell proliferation, differentiation, apoptosis, and stress resistance [26,27]. Studies have also shown that aberrant miRNA expression is correlated with the development and progression of cancer, thus miRNAs could be used as biomarkers for diagnosis and prognosis of cancer. On the other hand, the miRNAs can have oncogenic or tumor suppressor activities, so miRNAs are emerging as targets for cancer molecular therapy [28].

Extensive profiling studies over the past several years have shown that various miRNAs are differentially expressed in HCC and other types of cancers [29-41]. Nevertheless, there is still a lot remaining to be understood in the involvement of miRNAs in hepatocarcinogenesis and progression of HCC. Among all the HCC-related miRNAs, miR-221 was reported to be increasingly expressed in HCC, compared with nondiseased and adjacent benign liver tissues [37,40,42-47]. However, the relationship between the miR-221 expression and clinicopathological parameters in HCC was not fully understood.

In the present study, we investigated the expression of miRNA-221 in HCC and their matched adjacent noncancerous liver tissues in formalin-fixed paraffin-embedded (FFPE) surgically resected samples. Furthermore, we analyzed the correlation between miR-221 level and different clinicopathological parameters of HCC. We also performed *in vitro* experiments to study the effect of miR-221 on the cell growth, cell cycle, caspase-3/7 activity and apoptosis in HCC cell lines Hep3B, HepG2 and SNU449.

## Results

### MiR-221 expression in HCC FFPE tissues and its clinical significance

**Figure 1:**
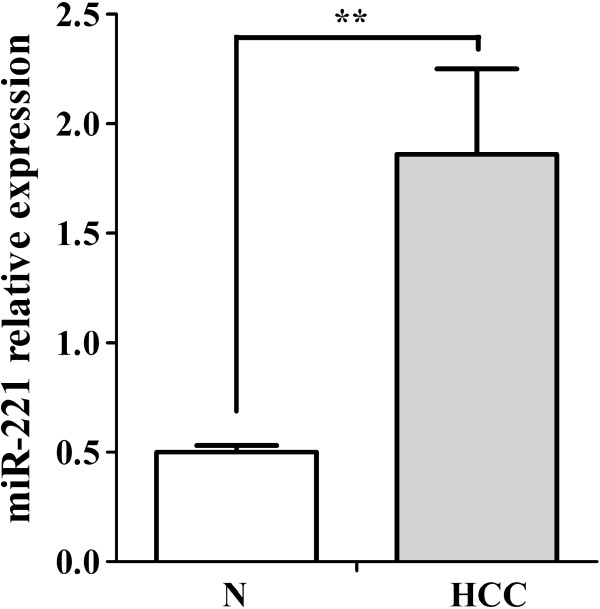
**MiR**-**221 relative expression in HCC FFPE samples.** MiR-221 levels accessed by real time RT-qPCR in HCCs and their adjacent noncancerous liver tissues. N: noncancerous liver tissues. ** *P*<0.01.

**Figure 2:**
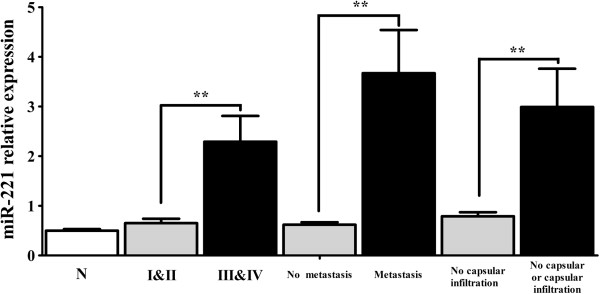
**Relationship of miR-221 relative expression and HCC clinicopathological features.** MiR-221 levels accessed by real time RT-qPCR in HCCs and their adjacent noncancerous liver tissues. N: noncancerous liver tissues. I&II: clinical TNM stage I and II, III&IV: stage III and IV. ** *P*<0.01.

**Figure 3:**
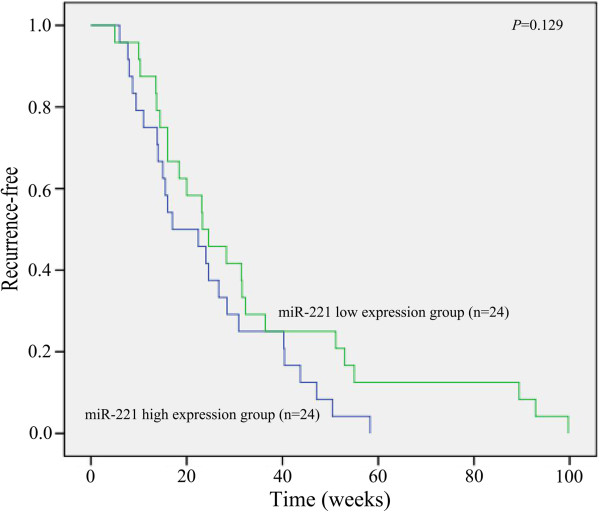
**Relationship between recurrence and expression of miR-221.** Forty-eight cases were followed up and the patients with high expression of miR-221 had a shorter time-to-recurrence compared to those with low expression.

The OD260/OD280 ratio of the total mRNA isolated from the FFPE tissues ranged from 1.84 to 2.06, and OD260/OD230 from 1.90 to 2.04. The PCR amplification efficiency of all the real time RT-qPCR reactions ranged from 91.0% to 95.2%. The relative expression of miR-221 in HCC tissues was significantly higher than that of their matched adjacent noncancerous liver tissues (*P*<0.05, Figure 1, Table 1). The expression of miR-221 in clinical TNM III and IV stages was also significantly higher than that in I and II stages. Furthermore, in the group with metastasis, miR-221 expression was upregulated compared to the group with no metastasis (*P*<0.05). When studied the relationship between miR-221 expression and other clinicopathological parameters, we found that miR-221 level was correlated with the status of tumor capsular infiltration. MiR-221 level became higher in the case that the tumors with capsular being infiltrated by cancer cells or the tumors with no capsular (*P*<0.05, Figure 2, Table 1). The miR-221 however had no correlation with other features, such as age, gender, histological differentiation grades, cirrhosis, plasma AFP levels, portal vein tumor embolus, number of the tumor nodes, tumor sizes or microscopic vaso-invasion (Table 1). Forty-eight among 76 patients were followed up and time-to-recurrence was collected. Time-to-recurrence for all 48 cases was 28.94±3.20 weeks. The patients with high expression of miR-221 (higher than the median level) had a shorter time-to-recurrence compared to those with low expression (24.15±3.11 vs 33.73±5.48), however, the difference is not significant (*P*=0.129, Figure 3).

**Table 1:** **Correlation between the expression of miR-221 and clinicopathological parameters in HCC **$\left(\bar{x}\pm s\right)$

**Clinicopathological parameters**		**n**	**miR-221 relavant expression**		
			***2***^***-△cq***^	***t***	***P***
Tissue	HCC	76	1.86±0.39	**3.46**	**0.001**
	Adjacent noncancerous liver	76	0.50±0.03		
Age	≥50	35	2.73±0.79	1.965	0.057
	<50	41	1.11±0.22		
Gender	Male	62	1.55±0.28	−0.965	0.351
	Female	14	3.25±1.74		
Differentiation	well	4	1.68±0.86	*F*= 1.487 *	0.233
	moderately	48	2.36±0.60		
	poorly	24	0.89±0.19		
Clinical TNM stage	I&II	20	0.65±0.09	**−3.098**	**0.003**
	III&IV	56	2.29±0.52		
Metastasis	Yes	31	3.67±0.87	**3.50**	**0.001**
	No	45	0.62±0.05		
With cirrhosis	Yes	40	2.01±0.66	0.402	0.689
	No	36	1.69±0.40		
AFP(μg/L)	≥400	30	1.84±0.81	−0.218	0.828
	<400	33	2.05±0.52		
Portal vein tumor embolus	Yes	18	3.63±1.45	1.582	0.131
	No	58	1.31±0.22		
Tumor capsular infiltration	no capsular or capsular infiltration	37	2.99±0.77	**2.856**	**0.007**
	no capsular infiltration	39	0.79±0.08		
Tumor nodes	multi	34	2.47±0.81	1.285	0.206
	single	42	1.37±0.26		
Tumor diameter(cm)	≥5	61	1.57±0.28	−0.947	0.36
	<5	14	3.24±1.75		
Vaso-invasion	Yes	19	3.67±1.37	1.738	0.098
	No	57	1.26±0.22		

* One-way Analysis of Variance (ANOVA) test was performed.

### MiR-221 inhibitor inhibits and miR-221 mimic enhanced cell growth in HCC cells

**Figure 4:**
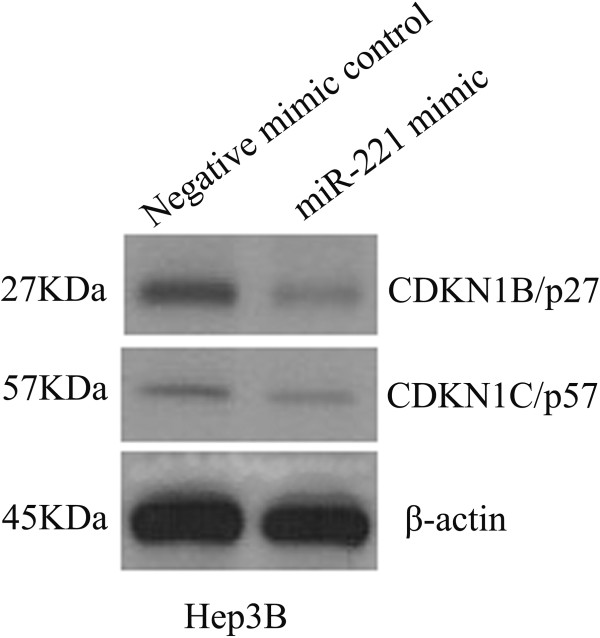
**Expression of CDKN1B/p27 and CDKN1C/p57 proteins after miR-221 mimic transfection.** HCC Hep3B cells were transfected with miR-221 mimic and negative control for 96 hrs, and the protein levels of CDKN1B/p27 and CDKN1C/p57 were performed using western blot.

To exclude effects of transfection efficiency of the transfection reagent on miR-221 effect, we determined HepB3 cells transfection efficiency with two approaches: cell fluorescence by siGLO Transfection Indicators and cell death induced by TOX Transfection Control. The transfection efficiency was higher than 89% at 72 hrs and 94% at 96 hrs, as assessed by either CellTiter-Blue® Cell Viability Assay, or fluorescence, as assessed by fluorescence microscopy (data not shown). These data suggested that the transfection efficiency was nearly optimal with the current method for transfection. Transfection efficiency of miR-221 mimic and inhibitor was further verified by RT-qPCR assay. The miR-221 expression level decreased with miR-221 inhibitor and increased with miR-221 mimic with a time-dependent manner, in different degrees. After transfection with the miR-221 inhibitor, the biggest ΔΔCq was 2.13 (77.15% knock-down) for HepB3, 1.32 (59.95% knock-down) for HepG2 and SNU449 1.78 (70.88% miR-221 knock-down) 96 hrs post-transfection. After transfecting the miR-221 mimic for 96 hrs, miR-221 levels were most severely increased, with ΔΔCq −13.78 (14065.74 folds upregulation) for HepB3, -12.44 (5555.65 folds upregulation) for HepG2 and −11.97 (4010.71 folds upregulation) for SNU449. Negative controls had no change of the level of miR-221 (data not shown). Furthermore, the protein levels of CDKN1B/p27 and CDKN1C/p57, known targets of miR-221 [47], were examined using western blot after different transfections. Indeed, downregulation of both CDKN1B/p27 and CDKN1C/p57 were observed when miR-221 mimic was transfected into HepB3 (Figure 4). Similar results also occurred in HepG2 and SNU449 cells (data not shown). On the contrary, an upregulation of CDKN1B/p27 and CDKN1C/p57 proteins were also seen after the transfection of miR-221 inhibitors (data not shown).

**Figure 5:**
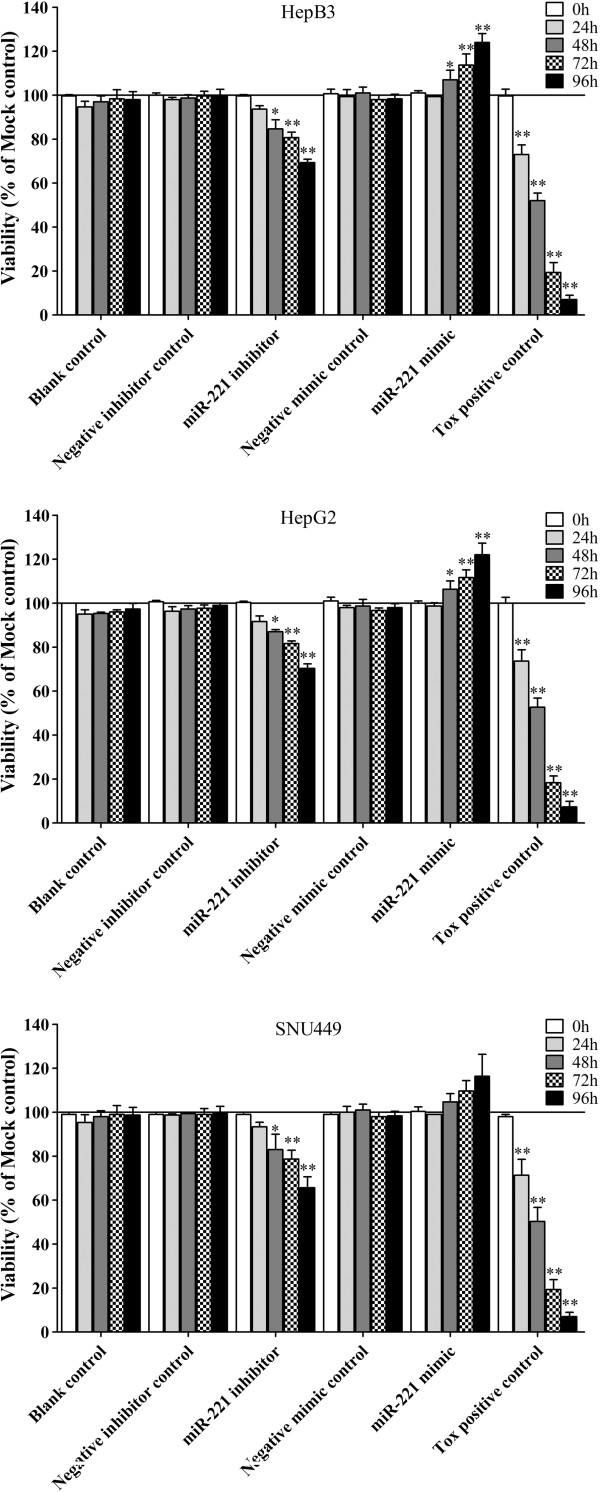
**Effect of miR-221 on cell viability of HCC cells by CellTiter-Blue Cell Viability Assay.** HCC cells were incubated in the presence of miR-221 inhibitor, mimic and different controls for 0, 24, 48, 72 and 96 hrs, and the cell viability was measured using the CellTiter-Blue Cell Viability Assay. * *P*<0.05, ** *P*<0.01, compared to blank control at the same time point.

**Figure 6:**
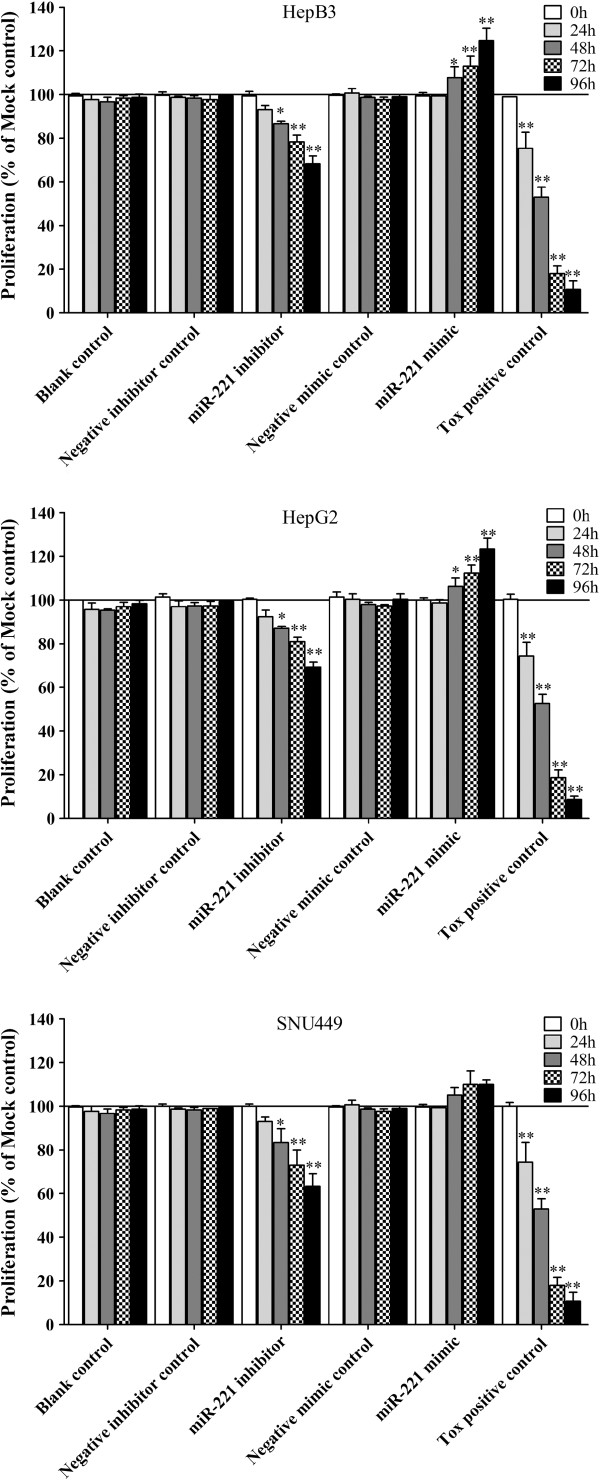
**Effect of miR-221 on cell proliferation of HCC cells by CellTiter96 AQueous One Solution Cell Proliferation Assay.** HCC cells were treated the same as described in Figure 5 and the cell proliferation was measured using the MTS assay (CellTiter96 AQueous One Solution Cell Proliferation Assay). * *P*<0.05, ** *P*<0.01, compared to blank control at the same time point.

**Figure 7:**
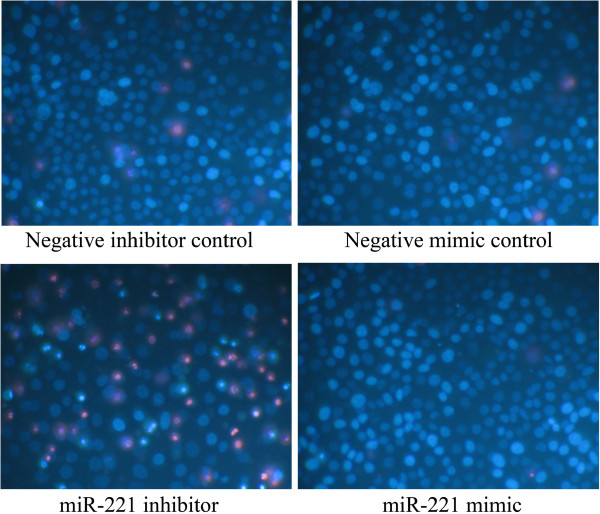
**Effect of miR-221 on cell growth and apoptosis of HCC HepB3 cells by Hoechst 33342/propidium iodide (PI) double fluorescent chromatin staining.** HCC HepB3 cells were transfected with miR-221 inhibitor, mimic and different controls for 96 hrs and the cells were observed under microscope with Hoechst 33342/PI double fluorescent chromatin staining, × 200.

**Figure 8:**
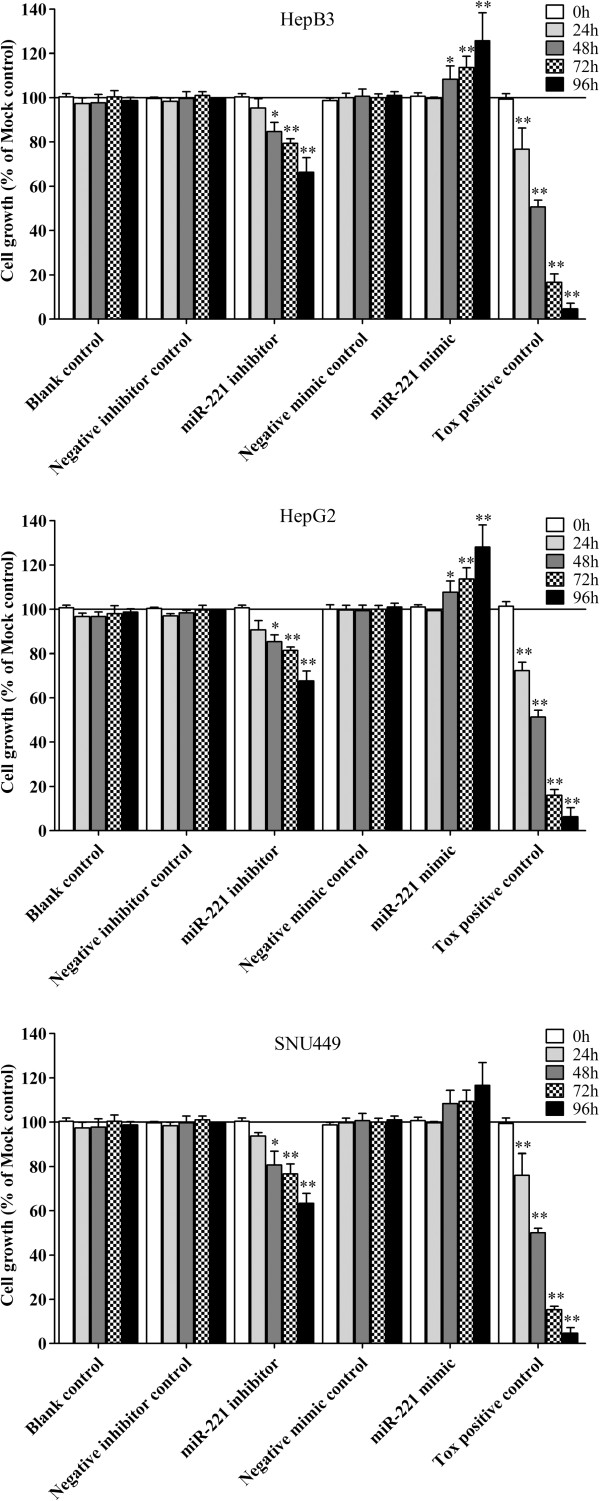
**Effect of miR-221 on cell growth of HCC cells by Hoechst 33342/propidium iodide (PI) double fluorescent chromatin staining.** HCC cells were treated the same as in Figure 5 and the cell growth was monitored with Hoechst 33342/PI double fluorescent chromatin staining. * *P*<0.05, ** *P*<0.01, compared to blank control at the same time point.

We then detected the effect of miR-221 on cell viability using a fluorimetric resorufin viability assay. With the miR-221 inhibitor, cell viability was significantly reduced in all cell lines tested from 48 hrs post-transfection compared to mock controls. After transfection with the miR-221 mimic, a significant increasing in viability was noted at the 48, 72 and 96 hrs in HepB3 and HepG2 compared to blank control. In SNU449 cells, the cell viability was slightly higher than the blank control, however, with no significant difference (Figure 5). To verify these results, the effect on cell proliferation was assessed using a MTS tetrazolium assay (Figure 6), and also by microscopic counting of viable (Hoechst 33342 positive/PI negative) cells (Figure 7, Figure 8). In both assays the results largely mirrored the fluorimetric resorufin viability assay results.

**Figure 9:**
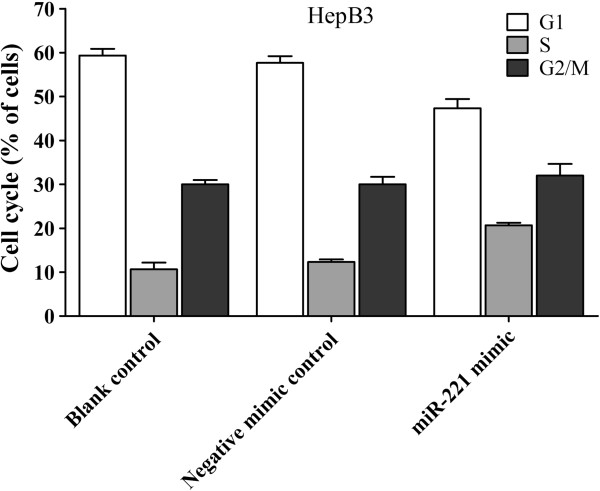
**Effect of miR-221 on cell cycle of HCC HepB3 cells.** HCC HepB3 cells were transfected with miR-221 mimic and negative mimic control for 96 hrs and the cell cycle was performed with flow cytometry.

Cell cycle analysis was further performed with HepB3 cells transected with miR-221 mimic and inhibitor. At 96 hrs post transfection, a 1.8-fold increase in the S-phase cell population was observed when compared to the blank control and the negative mimic control. Meanwhile, a concomitant decrease of the G1 phase population also occurred (Figure 9). Likewise, cell cycle was arrested in G1/S-phase by miR-221 inhibitor (data not shown).

### MiR-221 inhibitor induces cell apoptosis in HCC cells

**Figure 10:**
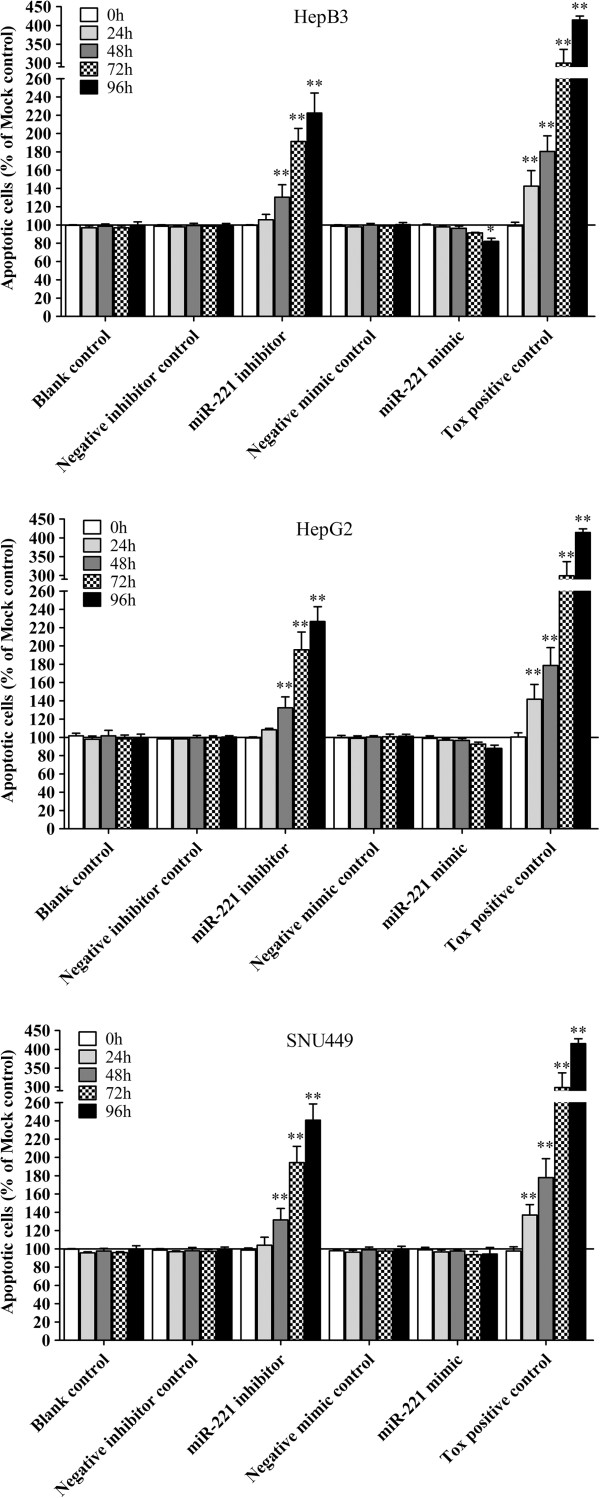
**Effect of miR-221 on apoptosis of HCC cells by Hoechst 33342/propidium iodide (PI) double fluorescent chromatin staining.** HCC cells were treated the same as above in Figure 5 and the apoptosis was monitored with Hoechst 33342/PI double fluorescent chromatin staining. * *P*<0.05, ** *P*<0.01, compared to blank control at the same time point.

**Figure 11:**
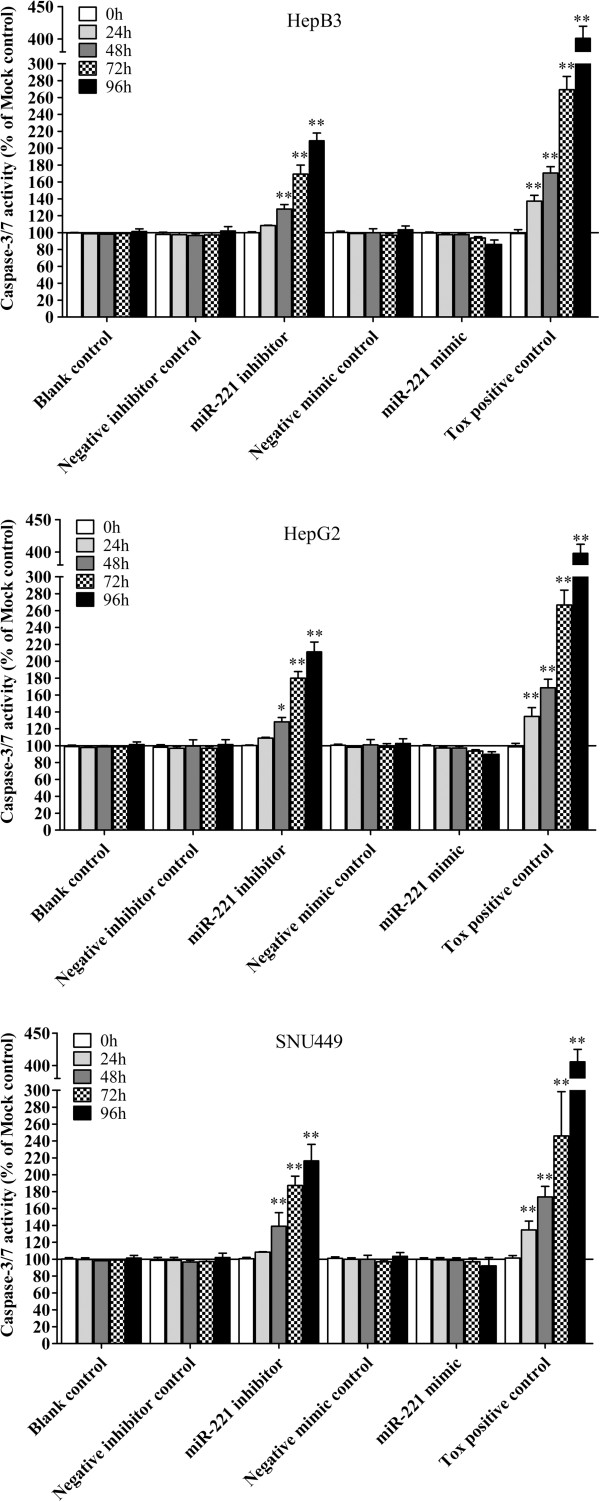
**Effect of miR-221 on caspase-3/7 activity of HCC cells.** HCC cells were treated the same as described in Figure 5 and the caspase-3/7 activity was detected using Apo-ONE Homogeneous Caspase-3/7 Assay. * *P*<0.05, ** *P*<0.01, compared to blank control at the same time point.

**Figure 12:**
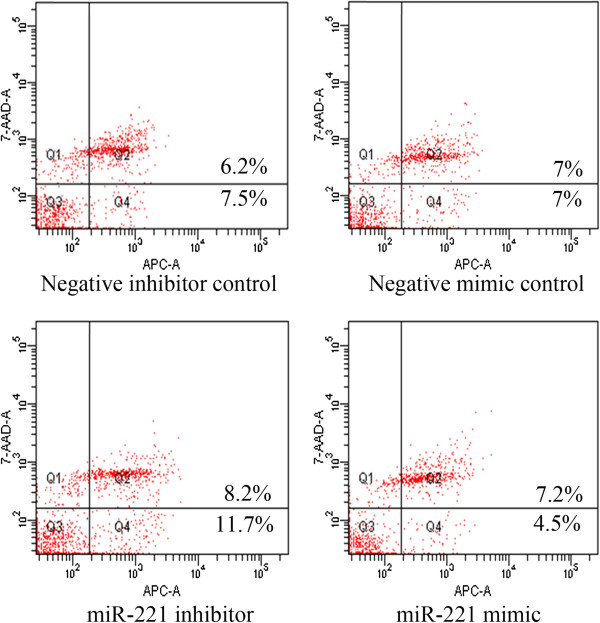
**Effect of miR-221 on apoptosis of HCC HepB3 cells by flow cytometry.** HCC HepB3 cells were treated with miR-221 inhibitor, mimic and different controls for 96 hrs and the apoptosis was accessed with 7-Amino-actinomycin D (7-AAD) / APC Annexin V staining by flow cytometry.

To verify whether miR-221 is able to influence apoptosis, the CellTiter-Blue assay was multiplexed with a fluorescent caspase-3/7 assay. The results showed that with the miR-221 mimic, caspase-3/7 activity was slightly less than the blank controls, but contained no significant change. However, with the miR-221 inhibitor, caspase- 3/7 activity was significantly enhanced in all three HCC cell lines tested (Figure 10). The effect on apoptosis was confirmed microscopically by Hoechst 33342 and PI double fluorescent staining (Figure 7, Figure 11). We also investigated the effect of miR-221 inhibitor and mimic on HepB3 cells with 7-Amino-actinomycin D (7-AAD)/APC Annexin V staining by flow cytometry (Figure 12). The results from flow cytometry was also consistent with those from caspase-3/7 assay and Hoechst 33342 and PI double fluorescent staining, which showed that with miR-221 inhibitor, apoptosis was induced , and on the contrary, miR-221 mimic attenuated apoptosis.

## Discussion

MiR-221 has been reported to be overexpressed in human HCC tissues, compared with normal liver tissues or adjacent benign liver tissues [37,40,42-48]. Four groups studied the miR-221 expression with fresh frozen samples using miRNA array, Northern blot or RT-qPCR assays [44,45,47,48]. FFPE tissues were used by only two groups to study the miR-221 expression in HCC. Fu et al.[43] used in situ hybridization (ISH) and real time RT-qPCR to detect the miR-221 level. Most recently, Karakatsanis et al. [42] also investigated the miR-221 expression with real time RT-qPCR in HCC FFPE tissues. In the current study, the result from real time RT-qPCR in 76 cases of HCC samples confirmed the previous reports, which showed that HCC had higher miR-221 level than other liver tissues. The overexpression of miR-221 in HCC indicates that miR-221 plays a critical role in the hepatocarcinogenesis, as an oncogenic miRNA.

Concerning the relationship between miR-221 expression and clinicopathological parameters, several groups reported that miR-221 level is related to the tumor TNM stages [42,43,45]. In the present study, miR-221 expression in stage III and IV was found to be significantly higher than that in stage I and II, which was in line with previous reports [42,43,45]. *In vivo* data also supported that miR-221 can promote tumor progression [44]. Meanwhile, consistent with Fu et al. [43], miR-221 expression was significantly upregulated in metastatic group compared to that in the nonmetastatic group. Additionally, miR-221 expression was correlated with the status of tumor capsular infiltration. In the group of tumor capsular infiltration with cancer cells or tumor with no capsular, miR-221 was significantly higher than that in the group of tumor without capsular infiltration. Generally, the status of tumor capsular infiltration reflects tumor invasion and metastasis. Thus, the result in current study reveals an obvious relation between miR-221 and the infiltration of tumor cells, migration, invasion and metastasis of HCC. The recurrence occurred quicker in the patients with high expression of miR-221 than those with low expression of miR-221. The difference was not significant, however there was a trend that high expression of miR-221 might lead to HCC recurrence. Hence it may be valuable to examine miR-221 expression for the clinical prediction of metastasis of HCC. The mechanisms of miR-221 promoting metastasis could be related to different targets. Garofalo et al. [48] showed that miR-221, by targeting PTEN and TIMP3 tumor suppressors, induced TRAIL resistance and enhance cellular migration through the activation of the AKT pathway and metallopeptidases. Besides the relationship between miR-221 and clinical stages, metastasis and capsular infiltration, Gramantieri et al. [45] reported that higher miR-221 levels were observed in multifocal HCCs versus unifocal tumors; miR-221 was also found to be corrected with tumor size[43] and cirrhosis [42]. Different from these reports, miR-221 expression in the current study was not correlated with the number of tumor nodes, tumor diameter or cirrhosis. The number of samples involved and different methods to detect miR-221 could partially explain the discrepancies. Additionally, miR-221 level was neither related to other clinical features, such as: age, gender, differentiation, AFP level or portal vein tumor embolus.

Recently, some miRNAs were also identified in serum and plasma in a remarkably stable form that is protected from endogenous RNase activity [49,50]. Li et al. [51] investigated the serum miR-221 expression in HCC. Similarly to HCC tissues, miR-221 was found to be differentially overexpressed in HCC serum samples, and high level of miR-221 expression was correlated with tumor size, cirrhosis and tumor stage. In addition, Kaplan–Meier survival analysis showed that the overall survival rate of the high miR-221 expression group (27.6%) was significantly lower than that of the low miR-221 expression group. Thus serum miR-221 could act as a noninvasive prognostic biomarker for HCC.

MiR-221 was also studied functionally *in vitro* and *in vivo*. Pineau et al. [44] investigated the role of miR-221 *in vitro*. After transfection of miR-221 precursor, they observed that most HCC cell lines, including HepG2 used also in our study, formed larger colonies than controls. When miR-221 antagomiR was transfected into HLE and Malhavu cells, the cell growth was drastically inhibited. Whereas, the same treatment led to no change in cell proliferation in PLC/PRF5 and Huh6 cell lines. In the current study, we transfected miR-221 inhibitor and mimic by combiMAGnetofection into different HCC cell lines (HepB3, HepG2 and SNU449). The cell growth was monitored by three independent assays: CellTiter96 AQueous One Solution Cell Proliferation Assay, CellTiter-Blue Cell Viability Assay and Hoechst 33342/PI double fluorescent chromatin staining. The results of the three methods were in agreement with each other and enhanced the observation of Pineau et al. [44]. With miR-221 inhibitor, the cell growth was inhibited in all the HCC cell lines tested. By contract, with miR-221 mimic, the cell growth was moderately accelerated in HepB3 and HepG2 cells. However, the effect of miR-221 mimic to enhance the cell growth in SNU449 cells was mild and without significantly difference when compared to the negative controls. Cell cycle was also detected by flow cytometry. When HepB3 cells were transfected with miR-221 mimic post 96 hrs, a 1.8 fold increase in the S-phase cell population was observed, which explained the character of miR-221 accelerating HCC cell growth. Yuan et al. [52] reported that miR-221 enhances proliferation of *in vitro* cultivated primary hepatocytes and adeno-associated virus mediated overexpression of miR-221 in the mouse liver also accelerates hepatocyte proliferation *in vivo*. Furthermore, miR-221 overexpression leads to rapid S-phase entry of hepatocytes during liver regeneration. These findings help explain the mechanism of the relationship between miR-221 and HCC cell proliferation. Fornari et al.[47] reported that cyclin-dependent kinase inhibitor (CDKN) 1C/p57 and CDKN1B/p27 are target genes of miR-221, and Yuan et al. [52] reported that Aryl hydrocarbon nuclear translocator (Arnt) mRNA can be a novel target of miR-221. Thus, overexpression of miR-221 can promote cell cycle progression, by affecting both CDKN1C/p57, CDKN1B/p27 and Arnt proteins.

There were contradictory reports on the relationship between miR-221 and apoptosis in HCC. Dai et al. [53] reported in HCC cells, endoplasmic reticulum (ER) stress-induced apoptosis is enhanced by miR-221 mimic and attenuated by miR-221 inhibitor. MiR-221 promoted-apoptosis under ER stress is associated with p27(Kip1)- and MEK/ERK-mediated cell cycle regulation. Thus, they concluded that suppression of miR-221 plays a crucial role in the protection against apoptosis induced by ER stress in HCC cells. On the contrary, Gramantieri et al. [37,45] found that the apoptosis of HCC cell line SNU449 was induced with knock-down of miR-221. Meanwhile, with a luciferase reporter assay, Bmf,a proapoptotic BH3-only protein [45], and DNA damage-inducible transcript 4 (DDIT4) [44], a modulator of the mTOR pathway, were confirmed to be targets of miR-221. Moreover, the analysis of HCC tissues revealed an inverse correlation between miR-221 and activated caspase-3, as a marker of apoptosis [45]. This explains that miR-221 can inhibit apoptosis by targeting Bmf and or/DDIT4. Furthermore, miR-221 has been identified as a potent posttranscriptional regulator of FAS-induced apoptosis [54] and necrosis factor related apoptosis-inducing ligand related apoptosis [55]. In the current study, Hoechst 33342/PI double fluerenscent staining observed by microscope and APC Annexin V/7-AAD staining by flow cytometry were performed to test the effect of miR-221 on apoptosis in HCC cells. The result of Gramantieri et al. [45] could be repeated in the present study. More than that, we also found that the apoptosis of HCC cell lines HepB3 and HepG2 was significantly increased with miR-221 inhibitor. However, miR-221 mimic did not succeed in reducing the apoptosis even with the concentration of miR-221 increasing to 300 nmol/L, suggesting that a saturation threshold was reached in these cell lines by a single miRNA mimic. To verify the data of apoptosis, we further detected the caspase-3/7 activity. The result of caspase-3/7 activity was in line with apoptosis. Hence, miR-221 could inhibit the apoptosis of HCC cells.

*In vivo* test has also been reported using the anti-miR-221 oligonucleotides as a potential therapeutic for HCC in mice [56]. Park et al. [56] showed that anti-miR-221 oligonucleotides could accumulate in the HCC tumors, reduce endogenous miR-221 oligonucleotides, modulate miR-221-related protein levels, and enhance the survival of tumor-bearing mice.

## Conclusions

Together with previous reports, the current observations strongly confirm that miR-221 is an oncogenic miRNA that plays a vital role in the initiation and progression of human HCC, by affecting multiple pro-oncogenic pathways. MiR-221 expression in HCC FFPE or sera samples could be a prognostic biomarker for HCC. On the other hand, cell growth inhibition and apoptosis induction by miR-221 inhibitor appears of great relevance due to its possible therapeutic role. The use of synthetic inhibitor of miR-221 might thus be a promising approach to HCC therapies in the future.

## Methods

### Patients

This retrospective study included 76 cases of HCCs and their corresponding paraneoplastic liver FFPE tissues. The ages of HCC patients ranged from 29 to 81 years old, with a mean of 52 years. A TNM classification (American Joint Committee on Cancer (AJCC)/International Union Against Cancer (UICC) staging system) was used to stratify HCC patients’ clinical stages [57]. This classification considers tumor size and number, vascular invasion, bilobar involvement and extra-hepatic metastasis. Clinicopathological information was obtained from medical records and summarized in Table 1. The corresponding paraneoplastic tissues were taken at least 2 cm from the cancerous node. All cases were initial hepatectomies and randomly chosen from the hepatectomies performed over a 1–2 year span in the First Affiliated Hospital, Guangxi Medical University, P.R. China between March 2010 and March 2011. Forty-eight patients were followed up till 6th, July, 2012. Time-to-recurrence was the time from randomization (operation date) to the time of radiological recurrence [58]. Study design was revised and approved by Guangxi Medical University Ethical Committee. Written informed consent to use the samples for research was obtained from the patients and clinicians. All samples were independently reviewed and diagnosed by two pathologists.

### Cell culture

The human HCC-derived cell lines HepB3 (ATCC HB-8064), HepG2 (ATCC HB-8065) and SNU449 (ATCC CRL-2234) were purchased from the American Type Culture Collection (ATCC, Rockville, MD,USA). HepB3 and HepG2 cell lines were cultured in Dulbecco’s modified essential medium (DMEM, Invitrogen Corp., Grand Island, NY, USA), whereas SNU449 was cultured with RPMI 1640. Both media were supplemented with 10% heat-inactivated fetal bovine serum (Invitrogen Corp., Grand Island, NY, USA), 2 mM glutamine, gentamicin, but without antibiotics, at 37°C in a humidified incubator with 5% CO_2_. All *in vitro* experiments were performed in triplicate.

### RT-qPCR

For clinical FFPE tissues, blocks were sectioned at a thickness of 10μm (3 sections for total RNA isolation). The tissues were dewaxed by xylene and ethanol. The total RNA was isolated from tumor sections using the miRNeasy FFPE Kit (QIAGEN, KJ Venlo, Netherlands) according to the manufacturer’s instructions with modifications by changing the incubation time after mixing with proteinase K to 36h at 55°C, meanwhile, adding proteinase K every 12 hrs to maintain its concentration. Depending on the size of the tumor sample, the RNA concentration ranged from 20ng/μl to 2μg/μl detected by Nanodrop 2000 (Wilmington, DE 19810 USA). Previously, we found the combination of RUN6B and let-7a was the most stable internal reference for HCC *in vitro* experiments by NormFinder and geNorm software, whereas the combination of RUN6B and RUN48 was the best housekeeping gene for HCC FFPE work (data not shown). Thus, different internal references were used in the current study. The primers for miR-221, RNU6B, RNU48 and let-7a were included in TaqMan® MicroRNA Assays (4427975–000468, Applied Biosystems, Life Technologies Grand Island, NY 14072 USA). The reverse primers were also used in the reverse transcription step with TaqMan® MicroRNA Reverse Transcription Kit (4366596, Applied Biosystems, Life Technologies Grand Island, NY 14072 USA) in a total volume of 10 μl. For *in vitro* experiments, the total Cellular RNA isolation was performed on the ABI PRISM 6100 prepstation using the AbsoluteRNA Solution (Applied Biosystems, Life Technologies Grand Island, NY 14072 USA) to remove contaminating DNA and PCR inhibitory substances. Real-time qPCR for miRNA was performed with Applied Biosystems PCR7900 [59-61]. The miR-221 abundance in each sample was normalized to its references. The miR-221 expression in FFPE experiment was calculated with the formula 2^-Δcq^, and the change ratio of miR-221 in the *in vitro* experiments was: (1-1/2^ΔΔCq^) × 100% [62].

### Re-expression and inhibition of miR-221 in HCC cells

HCC cells were seeded in a 24-well plate (2.5 × 10^4^ cells per well) or a 96-well plate (2.5 × 10^3^ cells per well) and incubated at 37°C for 24 hrs. The cells were then transfected with miR-221 inhibitor, miRNA inhibitor negative control, miR-221 mimic and miRNA mimic negative control (Ambion, Life Technologies Grand Island, NY 14072 USA) at a final concentration of 200 nmol/L using combiMAGnetofection (OZ BIOSCIENCES *,* Marseille cedex 9 France ) in accordance with manufacturer's procedure. The cells were transfected with the miRNA mimic or miRNA inhibitor daily up to 96 hrs. After transfection, intermediate samples at 24, 48 and 72 hrs were collected and analyzed by different assays.

### Western blot analysis

After transfection with miR-221 inhibitor, miR-221 mimic and different controls, the cells were washed with PBS and lysed in a buffer containing Tris/HCL (ph 7.6) 20mM, NaCl 150mM (ph 6.85), EDTA 1mM (ph 8), TRITON-X 1%, Na-pyrophosphate 2.5mM, Sodium orthovanadate (Na3VO4) 1mM, Leupeptin 1μg/ml, protease inhibitor cocktails 1% and phosphotase inhibitor cocktails 1% (Sigma-Aldrich N.V. St. Louis, USA). The lysates were centrifuged at 12,000 × g for 10 min at 4°C and boiled for 5 min. The protein concentration of the lysate was detected by the Bio-Rad Bradford protein assay and 25μg of denatured protein was subjected to SDS-PAGE (10% SDS-acrylamide gel) with a loading buffer containing 80mM Tris–HCl (ph 6.8), 5% SDS,10% glycerol, 5mM EDTA (ph 8), 5% 2-Mercapto Ethanol, 0.2% Bromophenolblue and 1mM phenylmethylsulfonyl fluoride The separated proteins were transferred to PVDF membranes (BioRad) for 2 hrs at 100 mA. The membrane was incubated with a p27 Kip1 (SX53G8.5) Mouse Monoclonal Antibody (#3698), p57 Kip2 Rabbit Polyclonal Antibody (#2557, 1:1000 dilution, Cell Signaling Technology, Inc.3 Trask Lane, Danvers, MA 01923) or a β-actin antibody (A1978 AC-15 1:2000 dilution, Sigma-Aldrich N.V. St. Louis, USA). Primary antibodies were detected with an HRP-conjugated secondary antibody (1:4000 dilution, ECL Anti-mouse or Anti-rabbit IgG Peroxidse linked Na 931, Sigma-Aldrich N.V. St. Louis, USA) and finally the membranes were subjected to chemiluminescence detection assay. Experiments were repeated in triplicate.

### Cell viability

Cell viability was assessed using a fluorimetric detection of resorufin (CellTiter-Blue Cell Viability Assay, G8080, Promega, Madison, USA). The protocol was as follows: miR-221 inhibitor, miR-221 mimic and their negative controls were transfected to 96 well plates and incubated at 37°C for up to 96 hrs. The procedure was according to the manufacturer. Fluorimetry (ex: 560 nm / em: 590 nm) was using an FL600 fluorescence plate reader (Bio-Tek, Virginia, USA). Fluorescence data are(or were) expressed as the fluorescence of treated sample / mock control ×100.

### Cell proliferation

To further confirm the data from the above cell viability assay, cell proliferation was detected by a colorimetric tetrazolium (MTS) assay (CellTiter96 AQueous One Solution Cell Proliferation Assay G3580, Promega, Madison, USA). The treatments and controls were as mentioned above. After transfections, addition of 20 μl of MTS reagent to each well, the plates were incubated for 2 hrs at 37°C in a humidified 5% CO_2_ atmosphere, and the absorbance at 490 nm was recorded using a 96-well microplate reader (Scientific Multiskan MK3, Thermo Finland). The results were the mean of six wells and expressed as the ratio of the absorbance of different transfections / absorbance of mock control × 100.

### Fluorescent microscopy evaluation of cell apoptosis and morphology

Besides the CellTiter-Blue cell viability assay and MTS assay, cell growth was also monitored with Hoechst 33342 (Sigma-Aldrich N.V. St. Louis, USA) and propidium iodide (PI, Sigma-Aldrich N.V. St. Louis, USA) double fluorescent chromatin staining. With this assay, the effects of miR-221 inhibitor and mimic on apoptosis and nuclear morphology in the HCC cells could also be assessed. In brief, after different transfections, cells were washed with ice-cold PBS and stained 15min with Hoechst 33342 (1 mg/ml) and PI (1 mg/ml), and observed under an advanced fluorescence microscope (ZEISS Axiovert 25, Munich, Germany). Apoptosis and nuclear morphology were identified by condensation of nuclear chromatin and its fragmentation. This system determines the absolute number of viable cells (Hoechst 33342 positive/PI negative), early apoptotic cells (Hoechst 33342 positive/PI negative with blue fragmentations in the cells), late apoptotic cells (Hoechst 33342 positive/PI positive, with red fragmentations in the cells), necrotic cells (PI positive) and debris signals. Viable, apoptotic and necrotic cells were counted in 10 different fields under the 200× vision in each well in three independent experiments by two persons and the average result was compared to the mock control. Apoptotic cell numbers from different treatments were compared by being normalized to their viable cell numbers [61].

### Flow cytometry analysis of cell cycle

HepB3 cells (1×10^5^) were selected to test the effect of miR-221 on cell cycle. Cells were plated into 6-well culture plates and treated with miR-221 inhibitor, mimic and their negative controls for 96h. Cells were collected with trypsin, then washed once with 4°C PBS, and fixed in cold 75% ethanol at 4°C. Cells were then washed once again with 4°C PBS and re-suspended with PBS, then stained with 50 mg/ml PI and 100 mg/ml RNase A solution (Genview, Carlsbad, CA) for 20 min at 37°C in dark. Stained cells were subjected to analysis immediately by flow cytometry. The proportion of cells in each phase of the cell cycle was determined by a BDFACScan for Quantitative Cell Analysis.

### Caspase-3/7 activity detection

Caspase-3/7 activity was measured using a synthetic rhodamine labeled caspase-3/7 substrate (Apo-ONE® Homogeneous Caspase-3/7 Assay, G7790, Promega, Madison, USA) performed immediately after the detection of cell viability (described above) on the same wells, according to the instructions of the manufacturer. After incubation at room temperature for 60min, the fluorescence of each well was measured (ex: 499 nm / em: 512 nm), using a FL600 fluorescence plate reader. Caspase-3/7 activity is expressed as fluorescence of treated sample / mock control×100.

### Flow cytometry analysis of apoptosis

HepB3 cells (1×10^5^) were also selected further to confirm the effect of miR-221 on apoptosis, using 7-Amino-Actinomycin (7-AAD)/APC Annexin V (BD Pharmingen™, South San Francisco, CA, USA) with flow cytometry. Cells were prepared as above and the procedure was according to the manufacturer. This assay allows to identify early apoptotic cells (7-AAD negative, APC Annexin V positive) and late apoptosis or already dead (both APC Annexin V and 7-AAD positive).

### Statistical analysis

SPSS19.0 (Munich, Germany) was used for statistical analysis. Results were representative of three independent experiments unless stated otherwise. Values were presented as the mean ± standard deviation (SD). One-way Analysis of Variance (ANOVA) test and Student’s paired *t*-test were used to analyze significance between groups. The Least Significant Difference (LSD) method of multiple comparisons with parental and control group was applied when the probability for ANOVA was statistically significant. Statistical significance was determined at a *P*<0.05 level.

## Abbreviations

HCC: Hepatocellular carcinoma; FFPE: Formalin-fixed paraffin-embedded; miRNAs: MicroRNAs; ICC: Intrahepatic cholangiocarcinoma; HBV and HCV: Hepatitis B and C virus; EGFR: Epidermal growth factor receptor; CDKN: Cyclin-dependent kinase inhibitor; DMEM: Dulbecco’s modified essential medium.

## Competing interests

The authors declare that they have no competing interests.

## Authors’ contributions

MHR, GC and YWD designed the study, performed statistical analysis and wrote the manuscript; GC performed the majority of experiments; YWD collected and cut the FFPE samples and sorted out pathological information. All authors read and approved the final manuscript.

## Pre-publication history

The pre-publication history for this paper can be accessed here:

http://www.biomedcentral.com/1471-2407/13/21/prepub
