# Correction to: Increased MiR-221 expression in hepatocellular carcinoma tissues and its role in enhancing cell growth and inhibiting apoptosis in vitro

**DOI:** 10.1186/s12885-020-6680-3

**Published:** 2020-03-16

**Authors:** Minhua Rong, Gang Chen, Yiwu Dang

**Affiliations:** 1 Research Department, Affiliated Cancer Hospital, Guangxi Medical University, 71 Hedi Road, Nanning, Guangxi Zhuang Autonomous Region 530021 People’s Republic of China; 2 Department of Pathology, First Affiliated Hospital, Guangxi Medical University, 6 Shuangyong Road, Nanning, Guangxi Zhuang Autonomous Region 530021 People’s Republic of China

**Correction to: BMC Cancer (2013) 13:21**

**https://doi.org/10.1186/1471-2407-13-21**

Following publication of the original article [1], the authors reported that they had misspelt the name of a cell line and supplied the incorrect Fig. 7 for publication.

The correct name of the cell line should be Hep3B, instead of HepB3. The correct figure is displayed below. The results and conclusions described therein are not affected by these corrections. The authors sincerely apologize for the error.

**Fig. 7:**
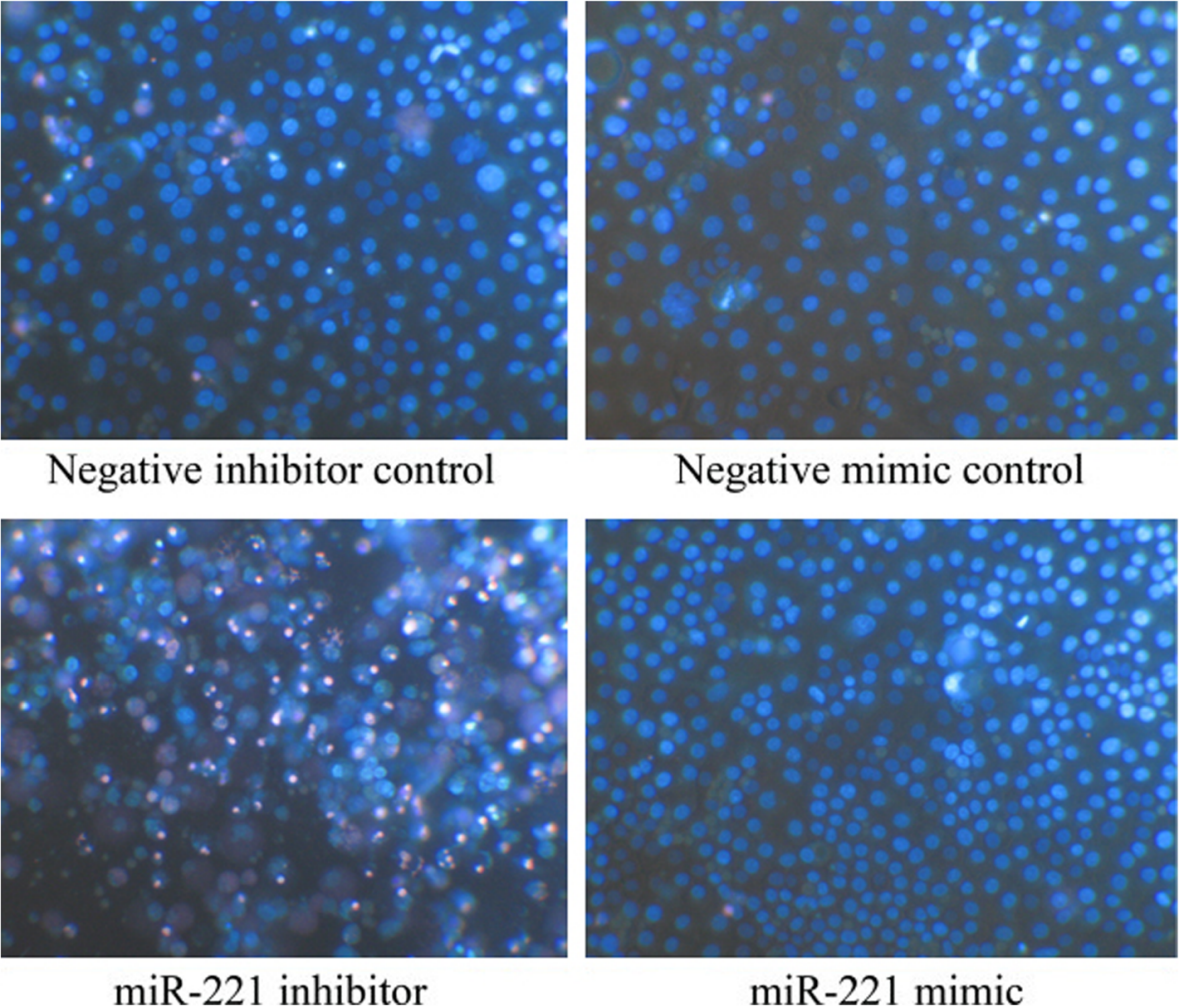
Effect of miR-221 on cell growth and apoptosis of HCC Hep3B cells by Hoechst 33342/propidium iodide (PI) double fluorescent chromatin staining. HCC Hep3B cells were transfected with miR-221 inhibitor, mimic and different controls for 96 h and the cells were observed under microscope with Hoechst 33342/PI double fluorescent chromatin staining, × 200
